# Conversion of Adipose-Derived Stem Cells into Natural Killer-Like Cells with Anti-Tumor Activities in Nude Mice

**DOI:** 10.1371/journal.pone.0106246

**Published:** 2014-08-27

**Authors:** Hongxiu Ning, Hong-En Lei, Yong-De Xu, Rui-Li Guan, Jeffrey M. Venstrom, Guiting Lin, Tom F. Lue, Zhongcheng Xin, Ching-Shwun Lin

**Affiliations:** 1 Knuppe Molecular Urology Laboratory, Department of Urology, School of Medicine, University of California San Francisco, San Francisco, California, United States of America; 2 Andrology Center, Peking University First Hospital, Peking University, Beijing, China; 3 Department of Medicine, School of Medicine, University of California San Francisco, San Francisco, California, United States of America; Hannover Medical University (MHH), Germany

## Abstract

Efforts to develop peripheral blood-derived nature killer (NK) cells into therapeutic products have been hampered by these cells' low abundance and histoincompatibility. On the other hand, derivation of NK-like cells from more abundant cell sources such as embryonic stem cells (ESCs) and umbilical cord blood (UCB) requires the selection of rare CD34+ cells. Thus, we sought to convert adipose-derived stem cells (ADSCs), which are abundant and natively CD34+, into NK-like cells. When grown in hematopoietic induction medium, ADSCs formed sphere clusters and expressed hematopoietic markers CD34, CD45, and KDR. Further induction in NK cell-specific medium resulted in a population of cells that expressed NK cell marker CD56, and thus termed ADSC-NK. Alternatively, the hematopoietically induced ADSCs were transduced with NK cell-specific transcription factor E4BP4 prior to induction in NK cell-specific medium. This latter population of cells, termed ADSC-NKE, expressed CD56 and additional NK cell markers such as CD16, CD94, CD158, CD314, FasL, and NKp46. ADSC-NKE was as potent as NK leukemia cell NKL in killing breast cancer cell MCF7 and prostate cancer cells DU145, PC3, LnCap, DuPro, C4–2 and CWR22, but exhibited no killing activity toward normal endothelial and smooth muscle cells. In nude mice test ADSC-NKE was able to significantly delay the progression of tumors formed by MCF7 and PC3. When injected into immunocompetent rats, ADSC-NKE was detectable in bone marrow and spleen for at least 5 weeks. Together, these results suggest that ADSCs can be converted into NK-like cells with anti-tumor activities.

## Introduction

Natural killer (NK) cells are an important component of the immune system [1]. Due to their ability to selectively kill target cells without prior sensitization, there have been intense interests to develop them into anti-cancer and anti-virus agents. In particular, their spontaneous cytotoxicity against a broad range of malignancies is a highly valuable attribute for their potential to become a “multi-purpose” anti-cancer agent. However, NK cells exist in the peripheral blood at a low number; therefore, after isolation they need to be expanded in culture to reach a sufficient number for clinical application. Nevertheless, prolonged culture leads to NK cell exhaustion; that is, the resulting cells become ineffective in killing target cells and die within a few days after infusion into the recipient [2]. Therefore, in recent years there have been attempts to generate NK cells from more abundant cell sources, such as embryonic stem cell (ESC) and umbilical cord blood (UCB) [3]–[8].

Adipose-derived stem cell (ADSC) is a type of mesenchymal stem cell that can be easily, safely, and abundantly obtained [9], [10]. While NK cell conversion from ESC and UCB requires pre-selection for rare CD34+ cells, ADSCs are natively CD34+ [11]–[13] and have been consistently shown to possess hematopoietic properties [14]–[21]. Thus, from a quantitative or qualitative standpoint, ADSCs represent a highly promising cell source for the generation of clinically applicable NK cells. In the present study we investigated the possibility of converting human ADSCs into NK-like cells that possess anti-tumor activities.

## Materials and Methods

### Primary cell isolation

Human ADSCs were isolated previously from the abdominal adipose tissue of a patient who underwent abdominoplasty [11]. Briefly, the adipose tissue was minced into small pieces, treated with collagenase, and the centrifuged. The resulting cell pellet was suspended in NH_4_Cl to lyse red blood cells, followed by centrifugation. The resulting pellet was suspended in Dulbecco's Modified Eagle's Medium (DMEM), filtered through a 40-μ strainer, and then stored in liquid nitrogen. In the present study, the frozen cells were thawed and seeded in DMEM-containing plastic culture dish. The attached cells were allowed to reach 80% confluence and then used for hematopoietic induction. Human cavernous smooth muscle cells (HCSMCs) and human cavernous endothelial cells (HCECs) were isolated previously from the corpus cavernosum of two separate patients who underwent penile prosthesis implantation [22]. Briefly, HCSMCs were isolated by tissue explant and HCECs by magnet beads coated with anti-CD31 antibody. The cells were cultured to 80% confluence, harvested, and then stored in liquid nitrogen. In the present study, the frozen cells were thawed, seeded in plastic culture dish, and then used for cytotoxicity assays.

### Cell cultures

Human umbilical vein endothelial cells (HUVECs) were purchased from Lonza Biologicals Inc. (Portsmouth, NH) and cultured in EGM2 medium (Lonza). Human prostate cancer cell lines PC3, DU145, LnCap, DuPro, C4–2 and CWR22 were kindly provided by Long-Cheng Li of University of California San Francisco) and cultured in RPMI-1640 medium supplemented with 10% fetal bovine serum (FBS) and 100 U/ml penicillin, 100 μg/ml streptomycin, and 0.25 μg/ml fungizone. Human leukemia cell line K562 and human breast cancer cell line MCF7 were purchased from American Type Culture Collection (Manassas, VA) and cultured as previously described [23]. Human NK leukemia cell line NKL was kindly provided by Lewis Lanier of University of California San Francisco and cultured in RPMI-1640 medium supplemented with 10% FBS and 12.5 ng/ml interleukin (IL)-2. ADSC-NK and ADSC-NKE were ADSC-derived NK-like cells generated in this study (see below) and were cultured in RPMI-1640 medium supplemented with 10% FBS, 12.5 ng/ml IL-2, 5 ng/ml IL-15, and 400 μg/ml G418 antibiotic (for E4BP4-transduced cells only).

### Hematopoietic induction

ADSCs (passage 0) were placed in 60-mm dishes containing a chemically defined, serum-free hematopoietic cell medium, X-VIVO 15 (Lonza), supplemented with 1% bovine serum albumin, 0.1 mM 2-mercaptoethanol, 200 μg/ml transferrin, 40 μg/ml low-density lipoprotein, 10 μg/ml insulin, 10 ng/ml thrombopoietin (Sigma-Aldrich, St. Louis, MI), 50 ng/ml stem cell factor (SCF), 50 ng/ml Flt3 ligand, 20 ng/ml granulocyte-macrophage colony-stimulating factor, 20 ng/ml IL-3, and 20 ng/ml IL-6 (R&D Systems, Minneapolis, MN). The incubation lasted for one week with the medium refreshed on days 3 and 6 while omitting IL-6 and thrombopoietin.

### Reverse transcription-polymerase chain reaction (RT-PCR)

Total cellular RNA was isolated from hematopoietically induced ADSCs and used for detection of hematopoietic markers by RT-PCR. The primer pairs used were as follows. CD34 (Forward: 5′-AAG CCT AGC CTG TCA CCT GGA-3′; Reverse: 5′-TGG CTT GCA ACA TCT TGC TCA-3′); CD45 (Forward: 5′-AGT GCA ACG TAA TGG AAG TGC-3′; Reverse: 5′-ACA GTT TCA TCC CTG GGA CCT-3′); CD105 (Forward: 5′-AAC ATG CAG ATC TGG ACC ACT-3′; Reverse: 5′-ACT GCG CAA GAC AAA CTT GTC-3′); KDR (Forward: 5′-TCA GCT ATG CTG GCA TGG TCT-3′; Reverse: 5′-TGG CTT GCA ACA TCT TGC TCA-3′). β-actin (Forward: 5′-TCTACAATGAGCTGCGTGTG-3′; Reverse: 5′-AATGTCACGCACGATTTCCC-3′) served as control.

### E4BP4 Transduction

Lentiviral packaging kit FPK-LvTR-20 and plasmid EX-T0508-Lv151, which is a lentiviral expression clone containing the protein-encoding sequence of human E4BP4, were purchased from GeneCopoeia (Rockville, MD). Packaging of EX-T0508-Lv151 into lentiviral particles and subsequent large-scale production of the E4BP4-containing lentivirus were done according to the user manual supplied with the packaging kit. Transduction of the E4BP4 lentivirus into hematopoietically induced ADSCs was also done according to the user manual. Expression of E4BP4 in transduced cells was verified by western blot with anti-E4BP4 antibody (Abcam, Cambridge, MA). β-actin control was probed with anti-β-actin antibody (Sigma-Aldrich). Western blot procedure has been described previously [24].

### NK cell induction

Hematopoietically induced ADSCs with and without E4BP4 transduction were trypsinized into single cells and transferred to T25 flasks containing RPMI-1640 supplemented with 50 ng/ml SCF, 50 ng/ml Flt-3 ligand, 12.5 ng/ml IL-2, 20 ng/ml IL-7, 40 ng/ml IL-15 (R&D), 2 mM glutamine, and 10% FBS. The incubation lasted for 4 weeks with the medium refreshed every three days. The resulting cells are called ADSC-NK and ADSC-NKE for the un-transduced and transduced cells, respectively, and were used at passages 1–5 in subsequent experiments.

### Flow cytometric analysis

Cells (1×10^6^ cells) at passages 1–5 were fixed and permeabilized with a fixation and permeabilization solution (BD Biosciences, San Jose, CA) at room temperature for 10 min. After washing with phosphate-buffered saline (PBS) twice, the cells were resuspended in 3% FBS-PBS and aliquoted into 10 tubes at 1×10^5^ cells/100 μl. Antibodies were added at concentrations suggested by the suppliers' data sheet. After incubation on ice for 2 h, the cells were washed three times and then analyzed in a fluorescence-activated cell sorter (FACSVantage SE System, BD Biosciences). The resulting data were further analyzed with FlowJo software (Tree Star, Inc., Ashland, OR). Antibodies against CD3, CD16, CD45, CD56, CD94, CD314, and FasL were purchased from eBioscience, Inc. (San Diego, CA). Antibodies against CD34 and NKp46 were purchased from BD Biosciences. Antibodies against CD105, CD158, KDR, and granzyme B (GB) were purchased from US Biological, R&D Systems, Santa Cruz Biotechnology, and Abcam, respectively.

### Cytotoxicity assay

A well-established flow cytometry-based method [7] was used in 4 different experiments, with 6 prostate cancer cell lines, MCF7, HUVECs, HCECs, and HCSMCs serving as target cells (1×10^4^ cells per assay) and ADSC, ADSC-NK, ADSC-NKE or NKL as effecter cells. All primary cells used in these experiments were from passages 1 to 5. In the first experiment – a dosage test, DU145 cells were labeled with 10 μM carboxyfluorescein diacetate succinimidyl ester (CFSE, Invitrogen, Carlsbad, CA) and then mixed with ADSC, ADSC-NK, ADSC-NKE, or NKL at ratios of 1∶25, 1∶50, and 1∶100. In the second experiment, all 6 prostate cancer cell lines and MCF7 were labeled with CFSE and each mixed with ADSC, ADSC-NK, ADSC-NKE, or NKL at a ratio of 1∶25. In the third experiment, PC3, HUVECs, HCECs, and HCSMCs were labeled with CFSE and each mixed with ADSC, ADSC-NKE, or NKL at a ratio of 1∶25. After overnight incubation, each cell mixture was stained with propidium iodide (PI, Sigma-Aldrich) and then analyzed by flow cytometry. The ratio of PI-stained versus CFSE-stained cells was calculated as the percentage of dead cells. In the fourth experiment, PC3 cells were labeled with CFSE and mixed with ADSC, ADSC-NKE, or NKL at a ratio of 1∶25. At 30 min and next day each cell mixture was observed and photographed microscopically. The phase-contrast and fluorescent images were then superimposed digitally. Intact and lysed CFSE-labeled cells were counted from 60 randomly selected images for each cell preparation at each time point. Since judgment of cell lysis could be subjective, the counting was performed by a coauthor who was blinded to the identity of cell samples.

### Detection of NK cell activation

Two different procedures were performed to detect NK cell activation. In the immunofluorescence procedure, 3 groups of ADSC-NKE cells (as effector) at 1×10^5^ cells per group were employed. The first group was negative control with no treatment. The second group was positive control treated with 2.5 μg/ml of phorbol-12-myristate-13-acetate (PMA, Sigma-Aldrich) and 0.5 μg/ml of ionomycin (Sigma-Aldrich). The third group was experimental group treated with the addition of 1×10^4^ K562 cells (as activator). Six h later, the cells were stained with anti-CD107a antibody (Abcam) and FITC-conjugated secondary antibody. Nuclear stain was performed with 4′,6-diamidino-2-phenylindole (DAPI). The stained cells were examined and photographed under a fluorescent microscope.

In the flow cytometric procedure, the same 3 groups of ADSC-NKE described above were employed, but this time with the addition of the following groups: (1) PC3 cells were used as another activator, besides K562 cells, (2) ADSC was used as a negative effector control, and (3) NKL was used as a positive effector control. After 6 hours of incubation, 20 μl/ml of anti-CD107a antibody was added. Another hour later, 10 μg/ml brefeldin A (Sigma-Aldrich) and 6 μl of Golgistop (BD bioscience) were added. Five more hours later the cells were washed twice with PBS, fixed with 1% PFA, and analyzed by flow cytometry as described under “Flow cytometric analysis”.

### In vivo anti-tumor activity assay

All procedures involving animal testing were approved by Institutional Animal Care and Use Committee of the University of California San Francisco. PC3 cells were injected subcutaneously into twelve 2-month-old male nude mice (Charles River Laboratories, Wilmington, MA) at 1×10^6^ cells/mouse. One week later, six of these mice were each intravenously injected with 1×10^7^ ADSC-NKE cells, and all 12 mice were each intraperitoneally injected with 10,000 U of IL-2 and 10 ng of IL-15. Afterward, IL-15 was injected daily for one week, and IL-2 every 3 days for the entire course. Tumor size was measured with an electronic caliper at 1 week and 2 weeks after PC3 injection, and every three days thereafter. The tumor size was calculated as length (mm) × width^2^ (mm^2^) × 0.523. MCF7 cells were tested similarly but with the following differences: (1) 20 female nude mice were used, with 10 each serving as treated and untreated mice, respectively, and (2) ADSC-NKE cells were injected 2 weeks after MCF7 injection. After the last tumor measurement, mice were euthanized by CO_2_ asphyxiation.

### Tracking transplanted ADSC-NKE cells in immunocompetent recipients

ADSC-NKE cells were labeled with thymidine analog 5-ethynyl-2-deoxyuridine (EdU) for 48 h [25] and then injected subcutaneously into thirty 4-month-old male Sprague-Dawley rats (Charles River Laboratories) at 1×10^6^ cells per rat. At each time point of 2 d, 1 wk, 2 wk, 3 wk and 5 wk, 6 rats were euthanized by intraperitoneal injection of 200 mg/kg pentobarbital and their bone marrow, spleen, lung, thymus, liver and kidney harvested for histology. All tissue samples were subjected to Click-iT reaction with azide-conjugated Alexa594 (Invitrogen) and DAPI staining to identify EdU+ cells and total cells, respectively [25].

### Statistical analysis

Data were analyzed using Prism 4 (GraphPad Software, San Diego, CA, USA) and expressed as mean ± standard error of mean (SEM). Multiple groups were compared using one-way analysis of variance followed by the Tukey-Kramer test for post-hoc comparisons. Statistical significance was set at p<0.05.

## Results

### Hematopoietic differentiation of ADSCs

**Figure 1:**
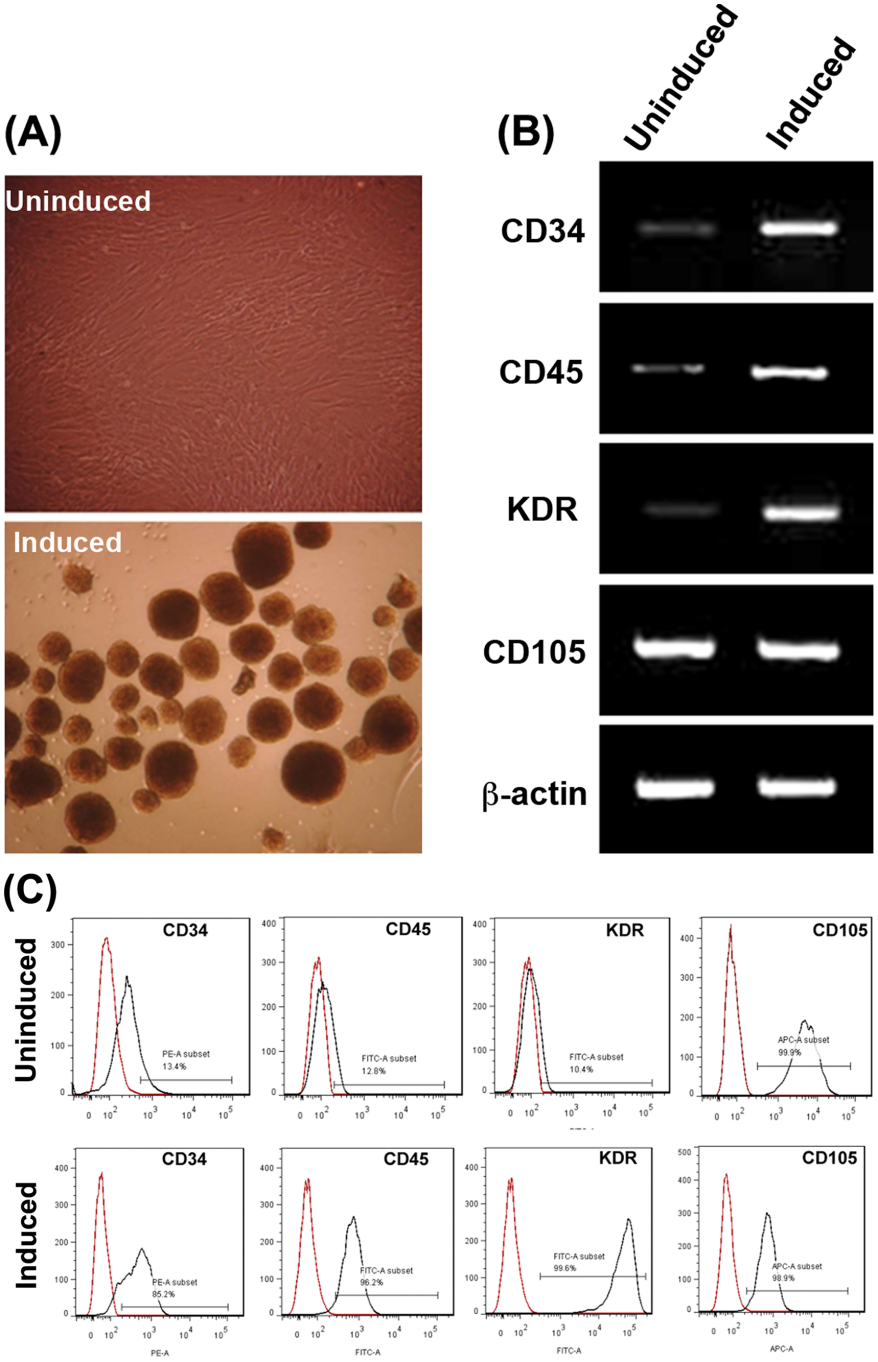
Hematopoietic conversion. After culturing in hematopoietic induction medium for one week, human ADSCs were photographed and then harvested for RT-PCR analysis. (A) The induced ADSCs detached from plastic surface and formed sphere clusters. (B) RT-PCR analysis showed that induced ADSCs expressed CD34, CD45, and KDR mRNA at much higher levels than un-induced ADSCs. (C) Flow cytometric analysis showed that induced ADSCs expressed CD34, CD45, and KDR cell surface antigens at much higher levels than un-induced ADSCs. Note that red lines indicate negative isotype controls for the respective antibodies. Each experimental and control group contained three replicates and each experiment was performed three times independently.

When cultured in hematopoietic induction medium for one week, ADSCs detached from plastic surface to form sphere clusters (Fig. 1A) and expressed CD34, CD45, and KDR at much higher levels than un-induced ADSCs (Fig. 1B&C). On the other hand, CD105, which was already expressed at a high level in un-induced ADSCs, remained highly expressed in induced ADSCs (Fig. 1B&C). By comparing the expression levels of CD34, CD45, and KDR between un-induced and induced ADSCs (13.4% vs. 85.2%, 12.8% vs. 96.2%, and 10.4% vs. 99.6%, respectively, Fig. 1C), the conversion rate was approximately 70 to 80%. For convenience, the hematopoietically induced ADSCs are called ADSC-HCs.

### Natural killer cell differentiation of ADSCs

**Figure 2:**
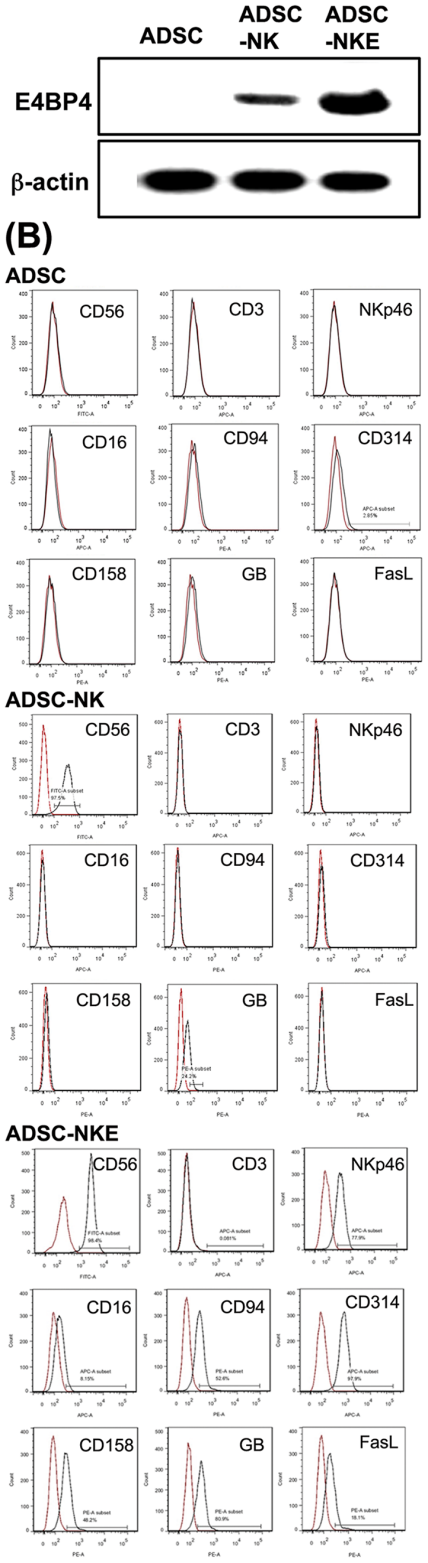
E4BP4 and NK cell marker expression. (A) Western blot analysis showed that ADSC had no detectable level of E4BP4 while ADSC-NK and ADSC-NKE had increasing levels of E4BP4. (B) Flow cytometric analysis for NK cell markers in ADSC, ADSC-NK, and ADSC-NKE. Note that red lines indicate negative isotype controls for the respective antibodies. Each experimental and control group contained three replicates and each experiment was performed three times independently.

To convert ADSC-HCs into NK cells, two different approaches were attempted. One was culturing ADSC-HCs in NK induction medium for 4 weeks; the other was transducing them with NK-specific transcription factor E4BP4 followed by 4 weeks of culturing in NK induction medium. Both resulting cell populations (called ADSC-NK and ADSC-NKE, respectively) proliferated with doubling time of 2 to 3 days (Fig. S1). While un-induced ADSC had no detectable level of E4BP4 expression, ADSC-NK and ADSC-NKE expressed incrementally higher levels of E4BP4 (Fig. 2A). Both ADSC-NK and ADSC-NKE also expressed high levels of CD56 but not CD3 (Fig. 2B). For all other tested markers, ADSC-NK expressed only GB at a detectable level whereas ADSC-NKE expressed each at levels ranging from 8.15% for CD16 to 97.9% for CD314. As for the un-induced ADSCs, CD314 was the only marker detectable (2.85%).

### Killing of cancer cells by ADSC-derived NK cells

**Figure 3:**
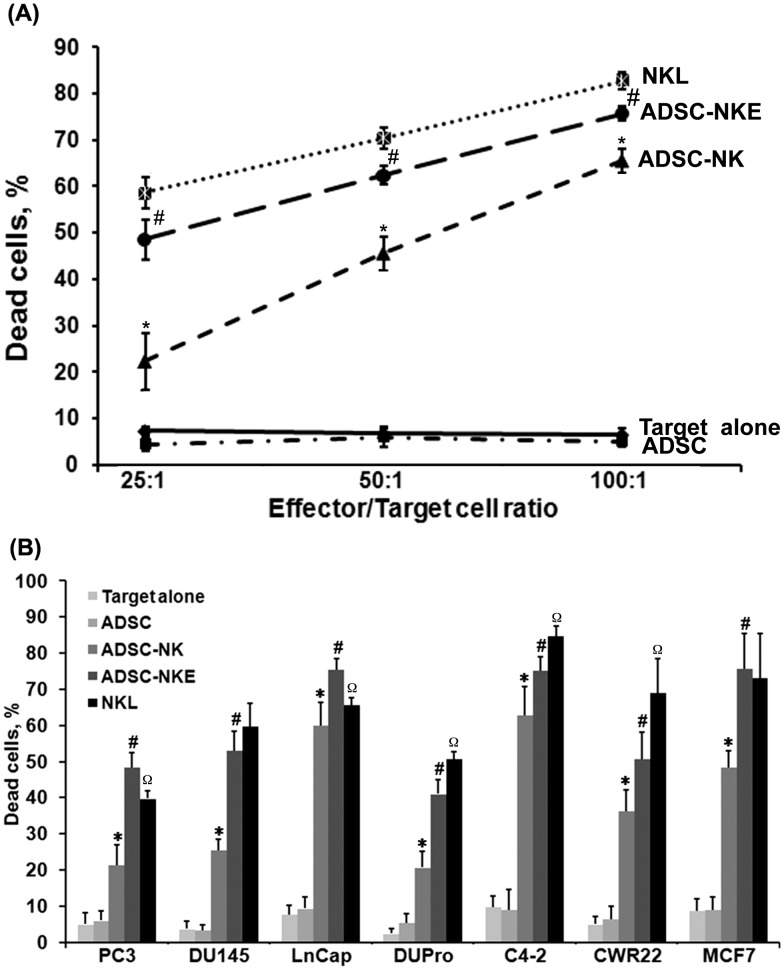
Cytotoxicity toward cancer cells. (A) ADSC, ADSC-NK, ADSC-NKE, and NKL were used as effector cells while prostate cancer cell DU-145 as target. Each effector was mixed with the target at the indicated ratio. The next day, each mixture was analyzed by flow cytometry to determine the ratio of dead versus live cells. * P<0.05, ADSC-NK versus ADSC; # P<0.05, ADSC-NKE versus ADSC-NK. (B) ADSC, ADSC-NK, ADSC-NKE, and NKL were used as effector cells while the indicated prostate cancer cell lines and MCF7 as targets. Each effector was mixed with the target at a ratio of 25∶1. The next day, each mixture was analyzed by flow cytometry to determine the ratio of dead versus live cells. * P<0.05, ADSC-NK versus ADSC; # P<0.05, ADSC-NKE versus ADSC-NK; Ω P<0.05, NKL versus ADSC-NKE. Each experimental and control group contained three replicates and each experiment was performed three times independently.

Whether ADSC-NK and ADSC-NKE were able to kill cancer cells was first tested against human prostate cancer cell line DU145, using a flow cytometry protocol for the detection of dead versus live target cells. The results showed that while ADSC had no killing activity, both ADSC-NK and ADSC-NKE exhibited increasing killing activities at increasing dosages, with ADSC-NKE being significantly more potent than ADSC-NK and nearly as potent as NKL (Fig. 3A). The test was then extended to include 5 additional prostate cancer cell lines, PC3, LnCap, DuPro, C4–2, CWR22, and breast cancer cell line MCF7 as targets. The results showed that while both ADSC-NK and ADSC-NKE were able to kill each of these 7 cancer cell lines, ADSC-NKE was significantly more potent than ADSC-NK in all instances and even more potent than NKL in certain instances (i.e., against PC3 and LnCap) (Fig. 3B). Thus, ADSC-NKE was chosen for further analyses.

### Activation, cytolytic activity, and cancer specificity of ADSC-derived NK cells

**Figure 4:**
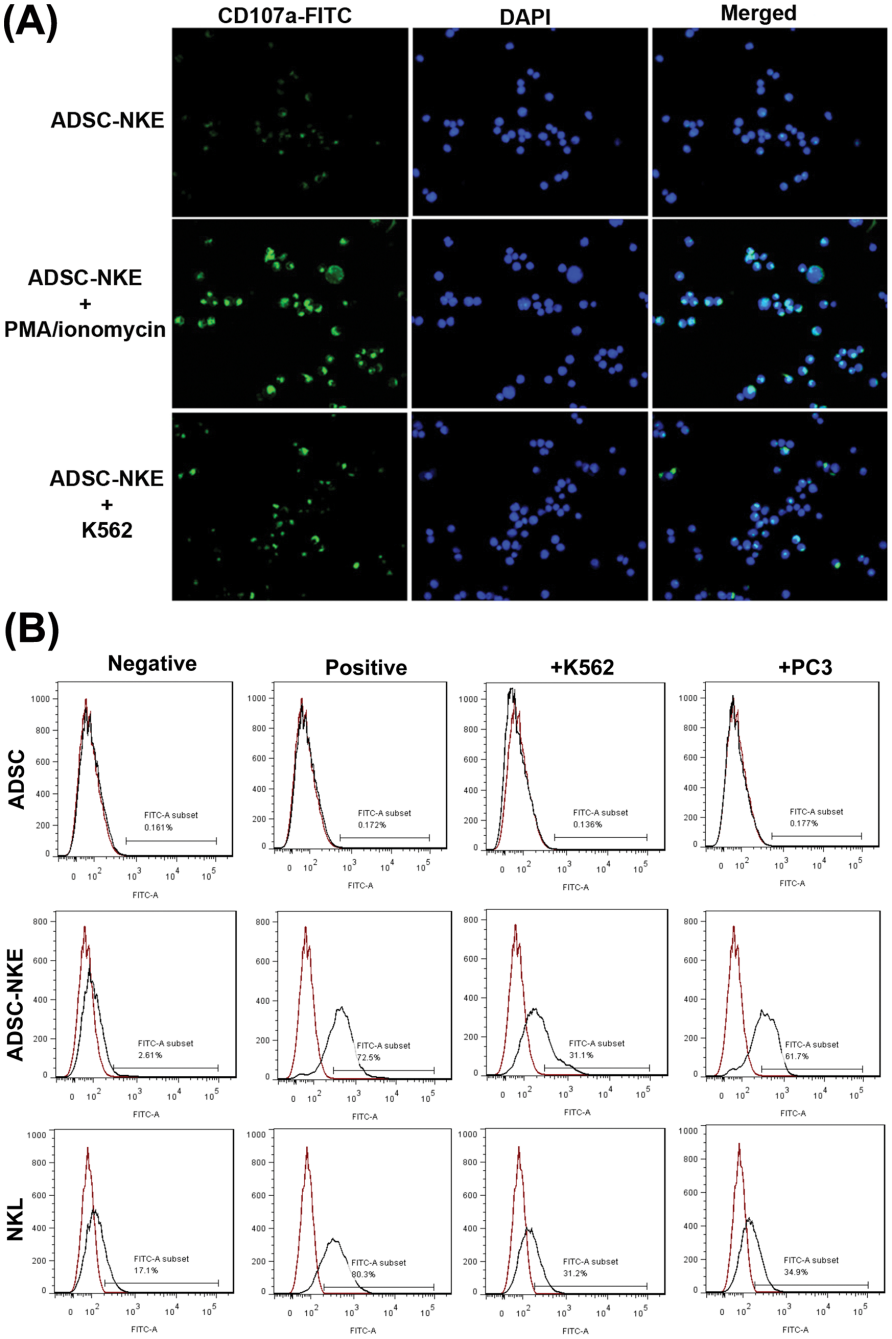
Cancer cell-induced activation. (A) ADSC-NKE cells were untreated (Negative control), treated with PMA/ionomycin (Positive control), or mixed with K562 cells at a ratio of 10∶1. Six hours later, the cell samples were stained for CD107a (green) and with DAPI (blue, for nuclei). (B) ADSC, ADSC-NKE, and NKL cells were untreated (Negative control), treated with PMA/ionomycin (Positive control), or mixed with K562 or PC3 cells at a ratio of 10∶1. Six hours later, the cell samples were analyzed for CD107a expression by flow cytometry. In the resulting histograms red lines indicate negative isotype control for anti-CD107a antibody. Each experimental and control group contained three replicates and each experiment was performed three times independently.

**Figure 5:**
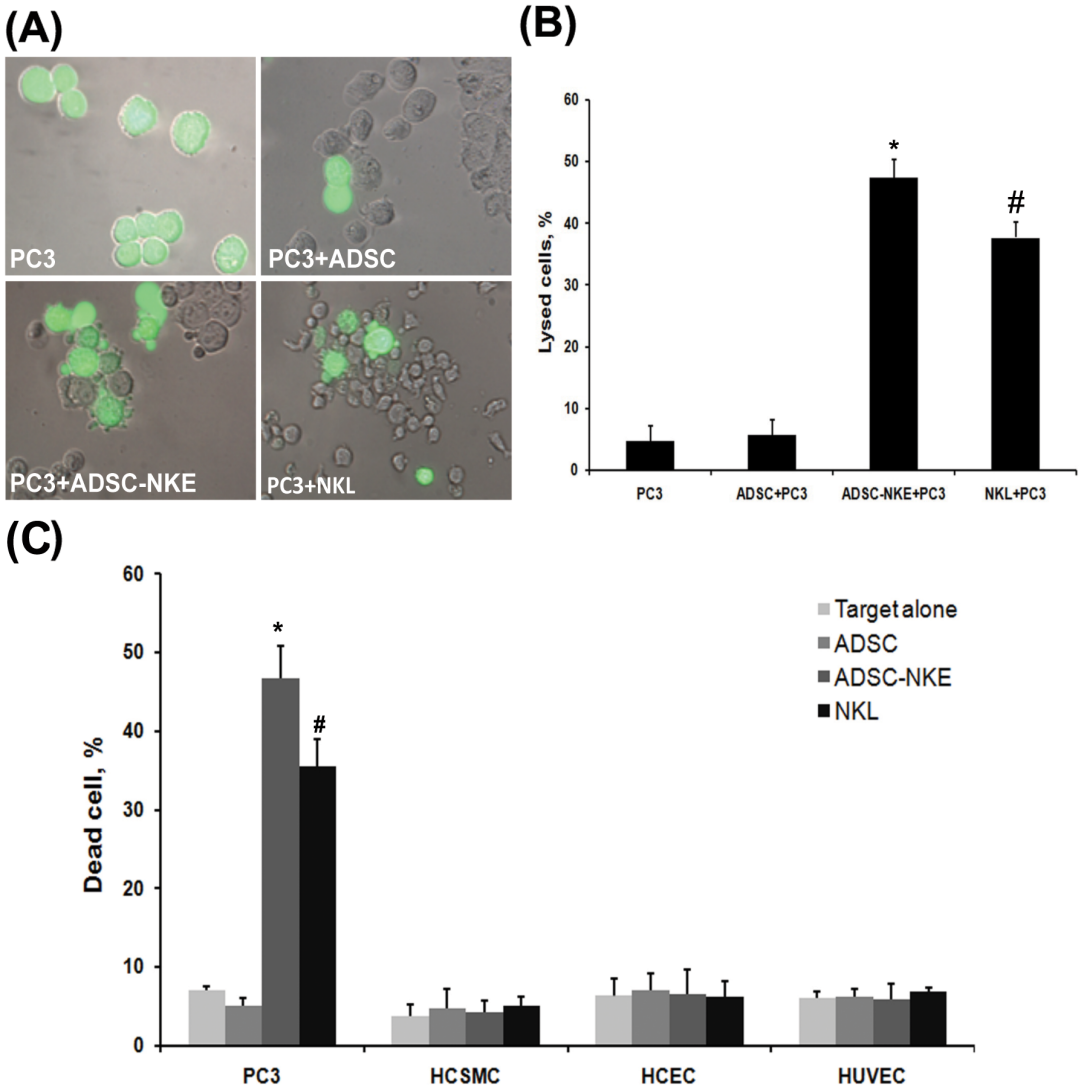
Cytolytic activities against cancer and normal cells. (A) ADSC, ADSC-NKE, and NKL were used as effector cells while prostate cancer cell PC3 as target. Each effector was mixed with the target (labeled with CFSE) at a ratio of 25∶1. After overnight incubation, each mixture was observed and photographed microscopically with phase-contrast and fluorescent settings. The phase-contrast and fluorescent images were then superimposed digitally. (B) From 60 randomly selected images for each cell preparation, intact and lysed CFSE-labeled cells were counted. The number of lysed cells was divided by the number of CFSE-labeled cells to generate the percentage of lysed cells. * P<0.05, ADSC-NKE versus ADSC; # P<0.05, NKL versus ADSC-NKE. (C) ADSC, ADSC-NKE, and NKL were used as effector cell while cancer cell PC3 and non-cancer cells (HCSMC, HCEC, and HUVEC) as targets. Each effector was mixed with each target at a ratio of 25∶1. The next day, each mixture was analyzed by flow cytometry to determine the ratio of dead versus live cells. Unmixed target cells served as controls. * P<0.05, ADSC-NKE versus ADSC; # P<0.05, NKL versus ADSC-NKE. Each experimental and control group contained three replicates and each experiment was performed three times independently.

Cancer cell-induced activation of ADSC-NKE was identified by increased cell-surface expression of CD107a when incubated with K562 leukemia cells and PC3 prostate cancer cells (Fig. 4). ADSC-NKE's ability to kill cancer cells by cytolysis was identified by the appearance of ruptured PC3 cancer cells (Fig. 5A&B). ADSC-NKE's specificity for killing cancer cells was demonstrated by the absence of killing activity toward non-cancer primary endothelial and smooth muscle cells (Fig. 5C).

### Anti-tumor activities of ADSC-derived NK cells

**Figure 6:**
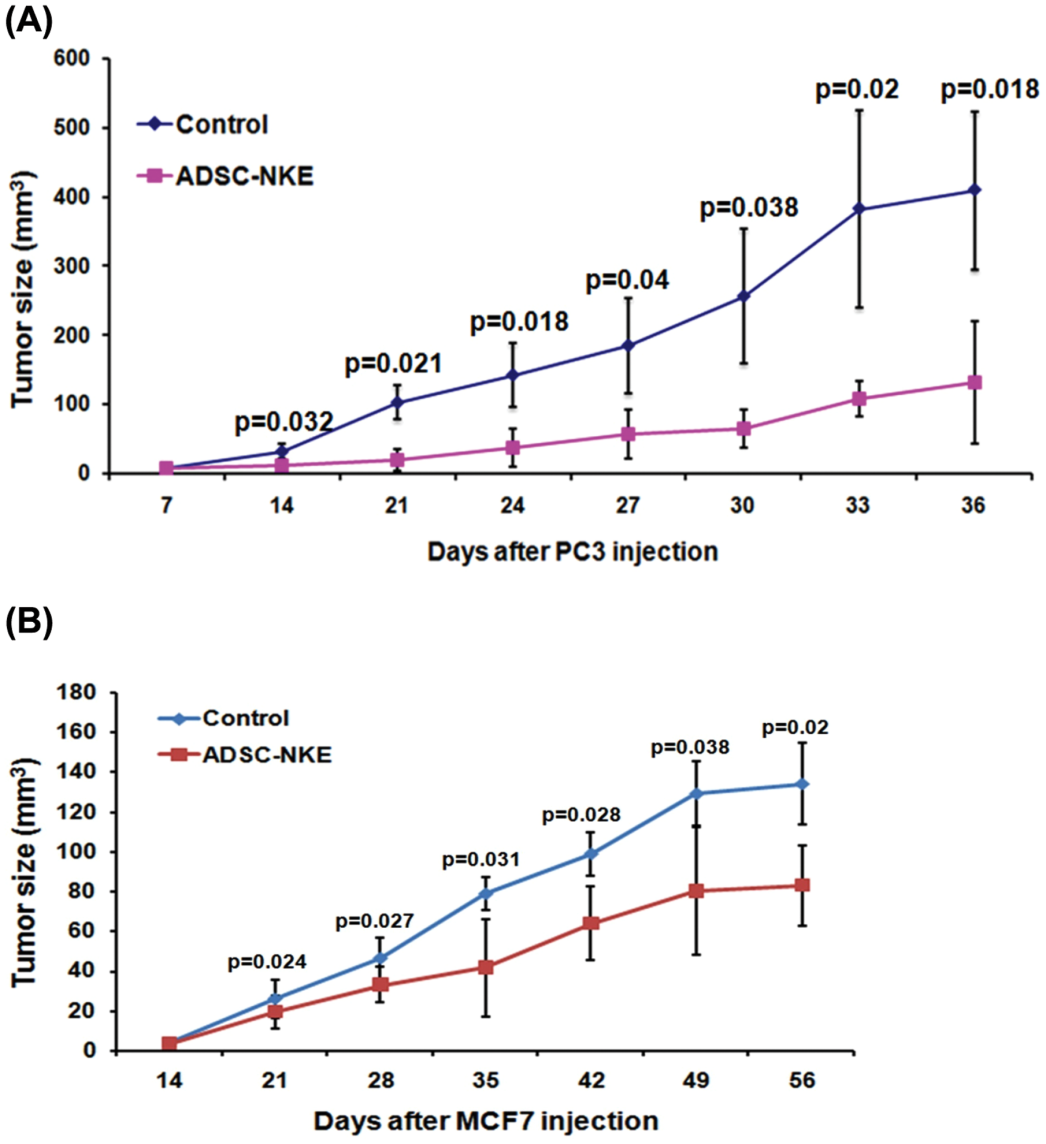
Anti-tumor activities. (A) Each of 12 male nude mice was subcutaneously injected with 1×10^6^ PC3 cells. One week later, 6 of these mice were each injected with 1×10^7^ ADSC-NKE cells via tail vein. All 12 mice were also each intraperitoneally injected with 10,000 U of IL-2 and 10 ng of IL-15. Afterward, each mouse received IL-15 injection daily for one week and IL-2 injection every third day for the entire course. Tumor size was determined at the indicated time points. (B) Same as in A except (1) 20 female nude mice were used, with 10 each serving as experimental and control animals, (2) MCF7 cells were used in place of PC3 cells, and (3) the ADSC-NKE and interleukin injections were initiated at 2 weeks after MCF7 injection.

ADSC-NKE's ability to delay tumor progression was demonstrated by the significant difference in PC3 or MCF7 tumor size between treated and control animals at each time point one week after the injection of ADSC-NKE cells (Fig. 6).

### Tissue distribution of ADSC-derived NK cells in immunocompetent recipients

**Figure 7:**
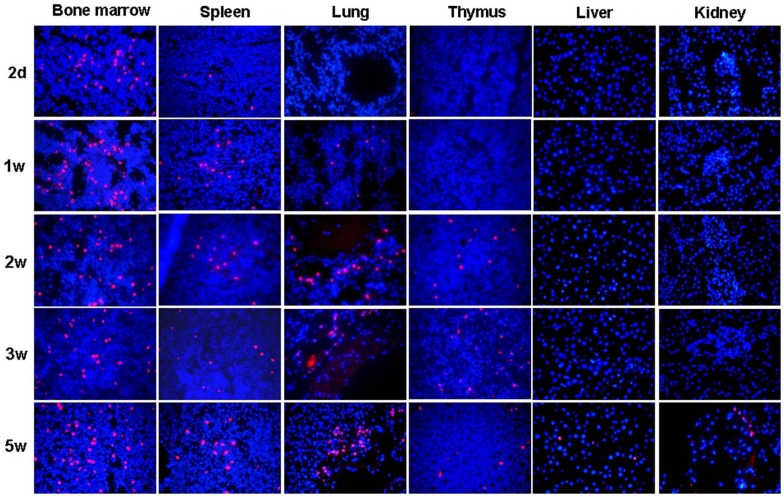
Survival and distribution of ADSC-NKE cells in immunocompetent recipients. EdU-labeled ADSC-NKE cells were injected subcutaneously into 30 Sprague-Dawley rats at 1×10^6^ cells per rat. At each indicated time point, 6 rats were sacrificed for tracking of EdU+ cells (red) in the indicated tissues as shown in the representative images (400×, with blue stains indicating cell nuclei).

After subcutaneous injection ADSC-NKE cells were detectable in bone marrow and spleen at all time points from 2 days to 5 weeks, in lung from 1 to 5 weeks, in thymus from 2 to 5 weeks, and in liver and kidney at the 5^th^-week time point only (Fig. 7).

## Discussion

Generation of cells that possess NK cell properties has been demonstrated previously with UCB cells and ESCs [3]–[8]. In all of these studies a CD34 selection step was implemented prior to NK conversion, and this is probably due to the low frequency of CD34+ cells in UCB and among hematopoietically induced ESCs. In the present study we subjected ADSCs to hematopoietic induction, but did not sort for CD34+ cells before NK conversion because the hematopoietic conversion appeared to have occurred at a high frequency as we observed in the induced ADSCs (1) a dramatic change of growth behavior from being adherent to non-adherent cells, and (2) a markedly increased expression of hematopoietic markers CD34 and KDR. Additionally, it should be noted that, similar to our present study, it has been reported that human adipose tissue contains a population of cells that formed sphere clusters when placed in non-adherent growth conditions [15]. This population is either the same or overlapping with ADSCs based on reports that consistently demonstrated the hematopoietic potentials of ADSCs [14]–[21]. Together, these studies indicate the strong hematopoietic propensity of ADSCs, and this in turn is probably because ADSCs are (1) CD34+ in their native tissue [11]–[13], and (2) capable of maintaining CD34 expression in culture when endothelial or hematopoietic growth factors are provided [15], [18], [19], [21], [24].

After successive hematopoietic and NK inductions, ADSCs became NK-like, as evidenced by the expression of CD56, but not CD3, and the ability to kill all 6 tested prostate cancer cell lines. However, this ADSC-NK cell population appeared to be sub-optimal as it expressed only one other positive NK marker (GB) at a detectable level, and when compared to the NKL cell line, it killed cancer cells less effectively. Therefore, we sought to generate a more potent NK cell population via the transduction of transcription factor E4BP4 into hematopoietically induced ADSCs.

E4BP4 is critical for the development and maturation of NK cells, as shown in two independent E4BP4-knockout studies [26], [27]. In these studies E4BP4-knockout mice were found to have normal numbers of B cells, T cells and NKT cells but almost no detectable NK cells [26], [27]. For those few detectable NK cells, their functionality was severely compromised as seen in their reduced production of interferon γ and cytolytic activity [26], [27]. Most importantly, transduction of E4BP4 into either knockout or wild-type hematopoietic progenitor cells was sufficient to increase NK cell production [26], [27].

ADSC-NKE cells, which harbored the transduced E4BP4, were similar to ADSC-NK cells in being CD56+CD3−. But, unlike ADSC-NK, ADSC-NKE expressed all of the tested positive NK cell markers. More importantly, ADSC-NKE was significantly more cytotoxic than ADSC-NK toward cancer cells, and, when compared to NK leukemia cell line NKL, ADSC-NKE was either equally or more cytotoxic against 3 out of 6 cancer cell lines. Thus, as far as in vitro cytotoxicity toward cancer cells is concerned, we consider ADSC-NKE to be functionally similar to true NK cells.

To further provide evidence for ADSC-NKE being NK-like, we investigated whether it could be activated by cancer cells. For this test we adopted a published procedure in which increased CD107a expression was demonstrated as a marker for NK cell activation [28]. Indeed, following either PMA/ionomycin treatment (positive control) or co-incubation with K562 cells, ADSC-NKE cells showed markedly increased CD107a expression. We also obtained direct visual evidence for ADSC-NKE's cytolytic activity against PC3 cancer cells, and we further showed that ADSC-NKE was able to distinguish between normal (endothelial and smooth muscle) and cancer cells. Together these data formed the basis for further testing ADSC-NKE's in vivo anti-tumor activities.

PC3 prostate cancer cell line has been frequently used in nude mouse-based tumorigenicity tests, including two of our previous studies [29], [30]. In these tests, PC3 cells at a dosage of either 1 or 2 million cells consistently formed subcutaneous tumors visible to the naked eye at 7 days post-injection. Therefore, we took this time point for starting the ADSC-NKE treatment. The results showed that, at 7-day intervals for the first two weeks and at 3-day intervals thereafter, the tumor size was significantly smaller in ADSC-NKE-treated than in untreated animals. In general, at any given time point, the treated mice's tumors were approximately 75% smaller than the untreated mice's. As for breast cancer cell line MCF7, which forms tumors much slower than PC3, significant differences between ADSC-NKE-treated and untreated mice was still evident at all tested time points. To our best knowledge, this is the first time in vivo anti-tumor activity has been demonstrated for converted NK-like cells. Note however that in vivo anti-leukemia and anti-myeloma activities have been demonstrated for ex vivo expanded NK cells [31], [32].

For clinical application, ADSC-derived NK-like cells will most likely be administered to patients who are immunocompetent, as opposed to the immunocompromised state as in the nude mice. Therefore, we investigated whether ADSC-NKE cells can survive in immunocompetent recipients by injecting ADSC-NKE cells in normal rats and then examining these rats' organs for the presence of the injected cells. The results showed that the injected cells were able to survive for at least 5 weeks, suggesting that ADSC-derived NK cells can be further investigated for use as an off-the-shelf multi-purpose anti-tumor agent regardless of the patient's HLA type.

## Supporting Information

Figure S1 **Cell proliferation.** ADSC-NK and ADSC-NKE were seeded in 96-well culture plates at 20,000 cells/well in 100 μl NK culture medium. At 0, 24, 48, 72 and 96 h, 20 μl of CellTiter 96 Aqueous One Solution reagent (Cat# G3580, Promega Inc., Madison, WI, USA) was added into each well. One h later the 96-well plate was scanned in a plate reader (Molecular Devices Corp., Sunnyvale, CA, USA) at 490-nm absorbance, followed by conversion of optical density values into cell numbers. The data are the average of three independent experiments.
(TIF)
