# Supplementary figures and images for: Conversion of Adipose-Derived Stem Cells into Natural Killer-Like Cells with Anti-Tumor Activities in Nude Mice
Source: PLoS One. 2014 Aug 27;9(8):e106246. doi: 10.1371/journal.pone.0106246 (PMC4146612; doi:10.1371/journal.pone.0106246)

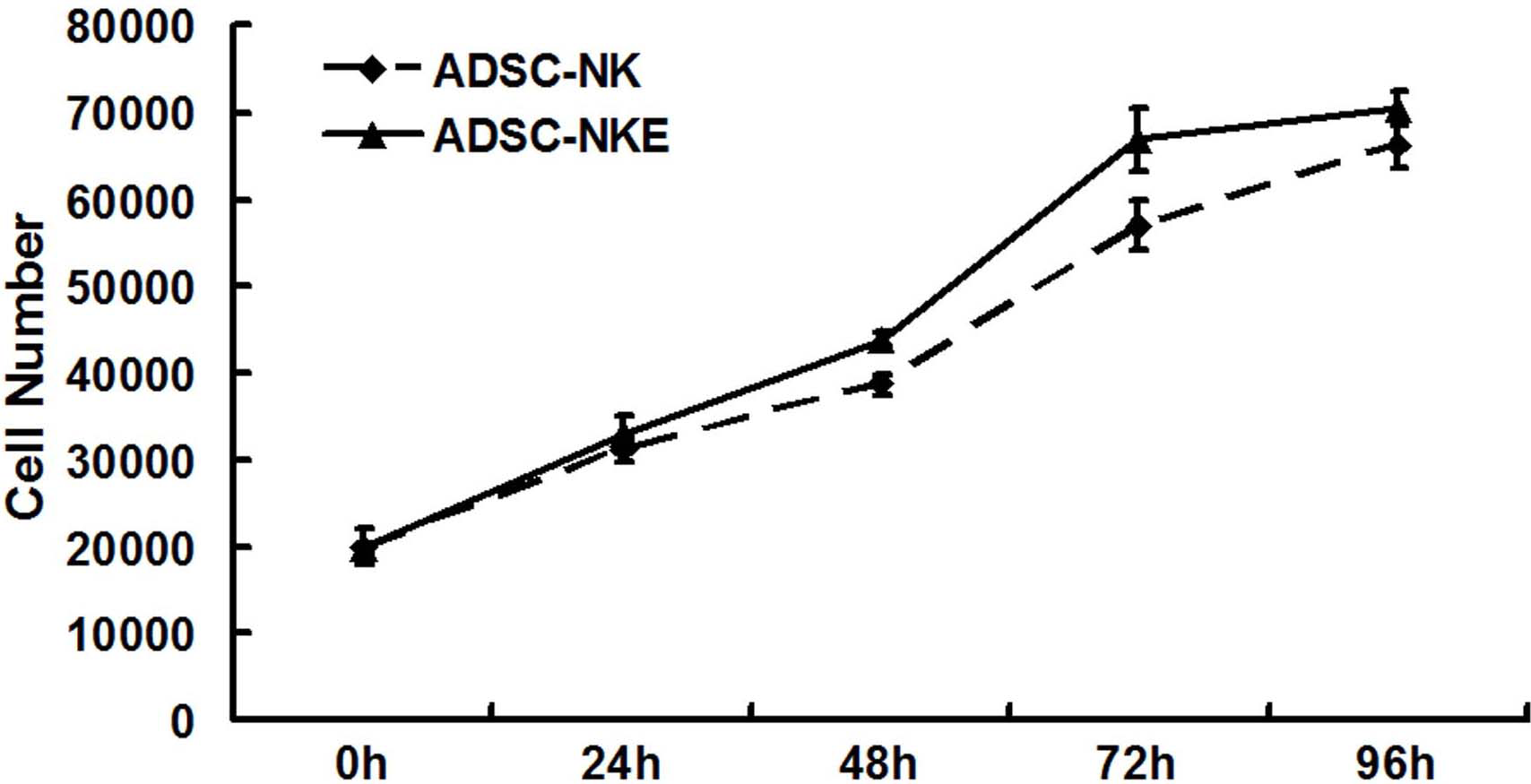

Supplement: Figure S1 — Cell proliferation. ADSC-NK and ADSC-NKE were seeded in 96-well culture plates at 20,000 cells/well in 100 μl NK culture medium. At 0, 24, 48, 72 and 96 h, 20 μl of CellTiter 96 Aqueous One Solution reagent (Cat# G3580, Promega Inc., Madison, WI, USA) was added into each well. One h later the 96-well plate was scanned in a plate reader (Molecular Devices Corp., Sunnyvale, CA, USA) at 490-nm absorbance, followed by conversion of optical density values into cell numbers. The data are the average of three independent experiments. (TIF) [file pone.0106246.s001.tif]
